# 基于三维重建技术的肺磨玻璃结节生长规律研究

**DOI:** 10.3779/j.issn.1009-3419.2023.101.11

**Published:** 2023-04-20

**Authors:** ZHOU Yingying, ZHANG Yongkui, ZHANG Shanhua, ZHANG Chi, CHEN Zhijun

**Affiliations:** ^1^154007 佳木斯，佳木斯大学（周莹莹，张弛）; ^1^Jiamusi University, Jiamusi 154007, China; ^2^316021 舟山，舟山医院胸外科（周莹莹，张永奎，陈志军）; ^2^Department of Thoracic Surgery; ^3^放射科（张善华）; ^3^Department of Radiology, Zhoushan Hospital, Zhoushan 316021, China

**Keywords:** 肺磨玻璃结节, 计算机断层扫描, 随访, 三维重建, Pulmonary ground glass nodules, Computed tomography, Follow-up, Three-dimensional reconstruction

## Abstract

**背景与目的** 自计算机断层扫描（computed tomography, CT）技术普及以来，影像学随访为主要管理方法的磨玻璃结节（ground glass nodules, GGNs）检出率明显增加，本研究旨在应用三维重建技术定量分析GGNs随访过程中的变化情况，探索GGNs自然进展规律，为临床指导患者合理地进行结节管理提供有效依据。**方法** 纳入2015年3月-2022年11月舟山医院肺结节联合门诊中规律随访的GGNs患者共115例。利用3D Slicer软件半自动分割提取结节的定量影像学特征，评估随访过程中的结节增长情况及临床干预情况。**结果** 患者平均基线年龄为（56.9±10.1）岁；平均随访时间为（48.8±18.9）个月。CT二维直径为（7.9±2.9）mm，三维最大径为（10.1±3.4）mm。末次CT扫描二维直径为（9.9±4.7）mm，三维最大径为（11.4±5.1）mm。共27例（23.5%）随访期间出现增长，中位体积倍增时间为822 d，中位质量倍增时间为1,007 d。手术切除32例，其中6例浸润性腺癌（invasive adenocarcinoma, IAC），16例微浸润腺癌（minimally invasive adenocarcinoma, MIA），8例原位腺癌（adenocarcinoma in situ, AIS），2例非典型腺瘤样增生（atypical adenomatous hyperplasia, AAH）。5例结节因二维直径显示进展而行手术干预，病理证实为浸润前病变，但其三维最大径提示无明显变化。单因素分析结果显示形态不规则、边缘欠光整，具有分叶、毛刺、空泡征均为促进结节增长的因素；增长组与稳定组年龄、基线直径和平均CT值、中位CT值、10%位数CT值、90%位数CT值差异具有统计学意义（P<0.05）。多因素Logistic回归分析结果表明年龄和平均CT值是影响结节增长的危险因素（P<0.05）。受试者工作特征（receiver-operating characteristic, ROC）曲线分析结果提示年龄≥63岁、基线三维最大径≥9.2 mm、平均CT值≥-507.8 HU的GGNs出现增长的可能性更大；三维最大径≥14.4 mm、平均CT值≥-495.7 HU时恶性概率更高。**结论** GGNs呈惰性生长过程，随访过程中应用三维测量值意义更大，对于持续存在的GGNs年龄≥63岁、基线三维最大径≥9.2 mm、平均CT值≥-507.8 HU出现增长的可能性更大，但多数结节出现进展后仍预后良好，长期随访是安全的。

## Abstract (en)

**Background and objective** Since the popularization of computed tomography (CT) technology, the detection rate of pulmonary ground glass nodules (GGNs) with imaging follow-up as the main management method has increased significantly. The purpose of this study is to quantitatively analyze the changes of pulmonary GGNs during the follow-up process with three-dimensional reconstruction technology, explore the natural progression of pulmonary GGNs, and provide effective basis for clinical guidance for patients to conduct reasonable management of nodules. **Methods** A total of 115 cases of pulmonary GGNs with regular follow-up in the Combined Outpatient Department of Zhoushan Hospital from March 2015 to November 2022 were enrolled. Quantitative imaging features of nodules were extracted by semi-automatic segmentation of 3D Slicer software to evaluate the growth of nodules and clinical intervention during follow-up. **Results** The average baseline age of the patients was (56.9±10.1) yr. The mean follow-up time was (48.8±18.9) months. The two-dimensional diameter of baseline CT scan was (7.9±2.9) mm, and the maximum three-dimensional diameter was (10.1±3.4) mm. The two-dimensional diameter of the last CT scan was (9.9±4.7) mm, and the maximum three-dimensional diameter was (11.4±5.1) mm. A total of 27 cases (23.5%) showed an increase during follow-up, with a median volume doubling time of 822 days and a median mass doubling time of 1,007 days. 32 cases were surgically resected, including 6 cases of invasive adenocarcinoma (IAC), 16 cases of minimally invasive adenocarcinoma (MIA), 8 cases of adenocarcinoma in situ (AIS) and 2 cases of atypical adenomatous hyperplasia (AAH). Five nodules underwent surgical intervention due to the progression of two-dimensional diameter, which was pathologically confirmed as pre-invasive lesions, but their three-dimensional maximum diameter showed no significant change. Nodular morphology, lobulated sign, spiculated sign and vacuole signs all promoted the growth of nodules in univariate analysis. There were significant differences in age, baseline diameter, mean CT value, median CT value, 10% and 90% percentile CT number between the growth group and the stable group (P<0.05). Multivariate Logistic regression analysis showed that age and average CT value were risk factors for nodule growth (P<0.05). Receiver-operating characteristic (ROC) curve analysis results indicated that the age ≥63 years old, the baseline three-dimensional maximum diameter ≥9.2 mm, and the average CT value ≥-507.8 HU were more likely to accelerate the growth of GGNs. The maximum three-dimensional diameter ≥14.4 mm and the average CT value ≥-495.7 HU may be a higher malignant probability. **Conclusion** GGNs show an inert growth process, and the use of three-dimensional measurements during follow-up is of greater significance. For persistent glass grinding nodules ≥63 years old, the baseline three-dimensional maximum diameter ≥9.2 mm, and the average CT value ≥-507.8 HU are more likely to increase. However, most nodules still have good prognosis after progression, and long-term follow-up is safe.

**【Competing interests】** The authors declare that they have no competing interests.

随着居民健康意识的提高和低剂量计算机断层扫描（computed tomography, CT）在体检中的广泛应用，健康体检人群的无症状肺磨玻璃结节（ground glass nodules, GGNs）检出率大幅提高^[1⇓-3]^，尤其是在非吸烟者中。肺部GGNs也已被认为是癌前病变或早期肺癌的可能征象。相较于实性结节的非小细胞肺癌，肺GGNs病理结果的恶性程度往往更低^[4⇓⇓-7]^。然而GGNs呈惰性生长的生物学行为及良好的预后与实体瘤相比存在显著差异^[8]^，因此，沿用以往实体瘤的诊治模式是不合适的，尤其是纯GGNs（pure GGNs, pGGNs），存在过度诊断及过度治疗的风险。磨玻璃密度影（ground glass opacity, GGO）为胸部CT中常见的影像学表现，是由肺泡内气体被液体与细胞成分置换所引起，导致局部肺组织的密度增高，在CT图像上表现为高于肺实质的密度，而又未掩盖肺纹理的高密度改变，且不伴肺门及纵隔淋巴结肿大、肺不张及肺炎等疾病^[9]^。形态学上分为pGGNs和部分实性结节（part-solid nodules, PSNs）^[10]^。可以是感染、出血、水肿及局灶性间质纤维化等良性病变所致；也可为原位腺癌（adenocarcinoma in situ, AIS）、微浸润腺癌（minimally invasive adenocarcinoma, MIA）等腺癌浸润前或浸润性病变^[11⇓-13]^；部分也可能是由坠积效应或呼吸运动伪影所导致的假磨玻璃样改变。目前围绕GGNs影像特征与肺腺癌病理亚型间相关性的研究较多，但揭示GGNs自然史的研究仍鲜少^[14]^。本研究分析2015年3月-2022年11月舟山医院肺结节联合门诊115例肺GGNs随访过程中的变化及干预情况，评估肺GGNs的自然进展规律，以期为临床指导患者合理进行结节管理提供有效依据。

## 1 资料与方法

### 1.1 研究对象

本研究纳入舟山医院2015年3月-2022年11月肺结节联合门诊中符合本研究标准的共115例GGNs患者。纳入标准：（1）患者年龄<80岁；（2）CT首次证实为GGNs后持续存在时间≥3个月；（3）GGNs三维最大直径≤2 cm；（4）CT纵隔窗实性成分长轴≤5 mm；（5）首次发现到末次随访时间间隔≥2年；（6）随访时间内未接受任何抗肿瘤治疗；（7）如果结节条件超过（3）或（4）但满足由于一般情况较差或个人原因不愿意接受手术干预强烈要求随访者；（8）CT检查图像无影响结节观察的运动伪影或金属伪影。

### 1.2 扫描及测量方法

采用GE LightSpeed 16和Toshiba Aquilion 64排CT机行常规胸部扫描。扫描参数：管电压120 kV，螺距0.993，矩阵768×768，CT层厚≤2.5 mm。肺窗窗位-500 Hu，窗宽1,500 Hu；纵隔窗窗位10 Hu，窗宽300 Hu。所有患者均在吸气末屏气后进行扫描，扫描范围自肺尖至肋膈角。所有随访CT图像均上传至3D Sliccer软件（www.slicer.org），手动分割辅助人工测量提取结节大小、CT值、表面积和体积。每个结节测量时设置灰度值最低阈值后逐层勾画，提取结节影像学定量参数（图1）。

**图1:**
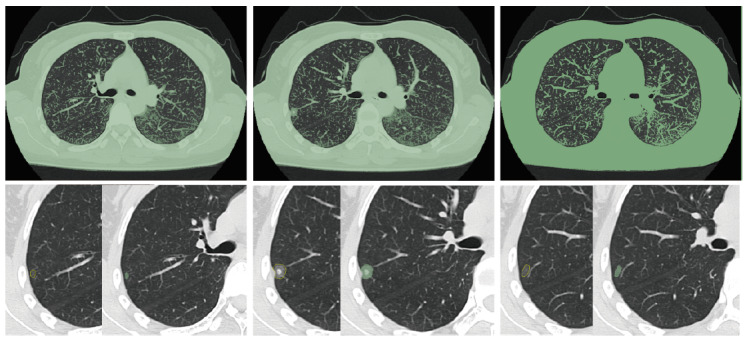
设置灰度值最低阈值后逐层勾画

### 1.3 临床资料及图像分析

患者的临床资料由1名医师收集和记录，包括性别、基线年龄（首次CT检查发现GGNs的年龄）、肺部基础疾病状态（肺炎、肺气肿等）。CT图像由2名中级职称医师采用双盲法分别阅片分析，误差≤2 mm时，取两读数的平均值；误差>2 mm时，由高年资医师重新阅片。分析的影像内容包括：GGNs类型[根据实性成分的有无分为pGGNs、异质性GGNs（heterogeneous GGNs, hGGNs）和PSNs]、单/多发性、大小、部位、形状、边缘状态（分叶、毛刺）、轮廓清晰与否、内部成分及邻近血管、支气管及胸膜牵拉征象。

### 1.4 评价指标

本研究中GGNs增长的定义采用现阶段临床常用的判断标准，具体为：（1）GGNs三维最大直径增加≥2 mm；（2）GGNs中出现新的实性成分。若发生pGGNs新出现实性成分的同时结节最大径减小或PSNs中实性成分最大径增加而结节最大径减小，均规定为结节增长。以CT值为0 HU为界限区别实性成分和磨玻璃成分^[15]^。质量计算采用M=V×（平均CT值+1,000）×0.001（M为质量，V为体积）公式，体积及质量倍增时间（volume/mass doubling time, VDT/MDT）采用Schwartz公式修正方法计算^[16,17]^。

### 1.5 统计学方法

采用SPSS 21.0软件进行数据分析。单样本K-S检验是否符合正态分布，符合正态分布的计量资料以均数±标准差表示，偏态分布的计量资料以中位数表示。分别应用卡方检验和独立样本t检验比较定性资料和定量资料，偏态分布资料则采用Mann-Whitney U检验进行分析，应用Logistic回归分析患者临床资料及影像学征象与结节进展的关系，预测影响结节增长的主要因素，绘制受试者工作特征（receiver-operating characteristic, ROC）曲线，计算曲线下面积并求得界值，检验风险指数预测结节进展的效度。P<0.05为差异有统计学意义。

## 2 结果

### 2.1 基线时结节特征

表1显示了基线时结节的一般情况，共115例结节，平均年龄（56.9±10.1）岁，中位年龄57岁，其中男性28例，女性87例，平均随访时间（48.8±18.9）个月。分为3组，pGGNs（不含实性成分）80例、hGGNs（实性成分仅肺窗可见）29例和PSNs（纵隔窗可见实性成分）6例。38例（33%）单发，77例（67%）多发；最常见于右上肺（44%）和左上肺（32%）；多为外周分布（79%）。

**表1:** 基线GGNs的一般情况

Characteristics	GGNs (n=115)	Growth group (n=27)	Stable group (n=88)	χ^2^/t	P
Gender				1.547	0.214
Male	28	9	19		
Female	87	18	69		
Age (yr)	56.9±10.1	62.1±9.4	55.3±9.8	3.172	0.002
Smoking status				0.222	0.638
Smoker	26	7	19		
Never smoked	89	20	69		
No. of GGNs				2.073	0.150
Solitary	38	12	26		
Multitary	77	15	62		
Classification				8.047	0.018
pGGNs	80	13	67		
hGGNs	29	11	18		
PSNs	6	3	3		
Position				3.915	0.418
Left upper	32	11	21		
Left lower	12	1	11		
Right upper	44	9	35		
Right middle	4	1	3		
Right lower	23	5	18		
Distribution				0.783	0.376
Peripheral (outer 2/3)	91	23	68		
Central (inner 1/3)	24	4	20		
Pulmonary basal state				0.714	0.870
Normal	85	21	64		
Pneumonia	13	2	11		
Emphysema	10	2	8		
Bullae pulmonary	7	2	5		
Follow-up (mon)	48.8±18.9	55.2±22.0	46.8±17.6	1.802	0.080

GGNs: ground glass nodules; pGGNs: pure GGNs; hGGNs: heterogeneous GGNs; PSNs: part-solid nodules.

结节基线状态影像学特征参数见表2，二维直径（7.9±2.9）mm，三维最大径（10.1±3.4）mm，表面积117.3 mm^2^，体积97.6 mm^3^，质量32.3 mg。平均CT值（-638.0±101.7）HU，中位CT值（-655.2±99.3）HU，10%位数CT值（-782.1±58.9）HU，90%位数CT值（-471.9±171.4）HU，23例有分叶征，16例有毛刺征，37例有空泡征，4例有胸膜牵拉。37例呈胸膜下分布，14例邻近叶间裂。

**表2:** 基线GGNs的影像学特征

Feature	GGNs (n=115)	Growth group (n=27)	Stable group (n=88)	χ^2^/Z/t	P
Shape				13.509	<0.001
Quasi-circular	83	12	71		
Irregularity	32	15	17		
Edge rounding				7.824	0.005
Yes	48	5	43		
No	67	22	45		
Clear outline				10.154	0.001
Yes	52	5	47		
No	63	22	41		
Adjacent pleura (≤1 cm)				9.675	0.002
Yes	37	11	26		
No	78	6	72		
Adjacent interleaf fissure				0.750	0.387
Yes	14	2	12		
No	101	25	76		
Air bronchogram				4.702	0.003
Yes	7	4	3		
No	108	23	85		
Lobular sign				6.401	0.011
Yes	23	10	13		
No	92	17	75		
Burr sign				6.401	0.011
Yes	16	6	10		
No	99	21	78		
Vacuole sign				4.126	0.042
Yes	37	13	24		
No	78	14	64		
Pleural drawing sign				1.271	0.259
Yes	4	0	4		
No	111	27	84		
2D diameter (mm)	7.9±2.9	9.1±3.6	7.6±2.6	2.384	0.019
3D diameter (mm)	10.1±3.4	11.4±3.8	9.7±3.2	2.317	0.022
Surface area (mm^2^)	117.3	132.3	106.0	-1.735	0.083
Volume (mm^3^)	97.6	126.1	91.4	-1.590	0.112
Mass (mg)	32.3	53.7	28.1	-1.887	0.059
Mean CT value (HU)	-638.0±101.7	-589.4±126.6	-652.9±88.4	2.432	0.020
Median CT value (HU)	-655.2±99.3	-608.9±127.8	-669.3±84.7	2.303	0.028
10% percentile CT value (HU)	-782.1±58.9	-752.9±78.9	-791.0±48.4	2.370	0.024
90% percentile CT value (HU)	-471.9±171.4	-397.2±192.6	-494.9±158.6	2.657	0.009

CT: computed tomography; Surface area, volume and mass do not follow normal distribution and are described in terms of median.

### 2.2 结节的增长情况

随访期间出现1例新发。末次随访时，pGGNs 70例、hGGNs 35例和PSNs 10例。11例pGGNs转变为hGGNs；4例hGGNs进展为PSNs。终末CT扫描结节二维直径（9.9±4.7）mm，三维最大径（11.4±5.1）mm，表面积169.3 mm^2^，体积178.9 mm^3^，质量59.8 mg。平均CT值（-589.4±150.5）HU，中位CT值（-611.7±140.3）HU，10%位数CT值（-777.4±70.5）HU，90%位数CT值（-385.3±216.8）HU。共27例（23.5%）出现增长（图2），其中有24例（20.9%）2年内二维直径增长超过2 cm，2年内三维最大直径增长大于2 cm的共14例（12.2%）。所有结节的表面积、体积及质量均出现不同程度增长。中位体积倍增时间为822 d，中位质量倍增时间为1,007 d。随访期间13例出现空泡征，9例出现毛刺征，14例出现分叶征，7例新出现胸膜牵拉。

**图2:**
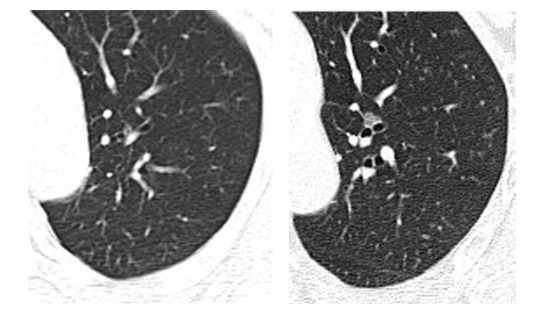
左上肺GGNs随访69个月后进展情况

### 2.3 切除结节的特征

32例结节行手术干预，2例肺叶切除，余均为肺段或楔形切除。切除pGGNs 14例，6例AIS，8例MIA；切除12例hGGNs，2例非典型腺瘤样增生（atypical adenomatous hyperplasia, AAH），2例AIS，6例MIA，2例浸润性腺癌（invasive adenocarcinoma, IAC）；切除PSNs 6例，MIA 2例，IAC 4例。15例单发，17例为多发。5例仅二维直径提示增长的GGNs病理均为MIA。切除结节CT测量二维平均直径为（11.5±5.5）mm，三维平均最大径为（13.6±6.3）mm，差异具有统计学意义（P<0.001）；病灶标本平均直径为（8.6±4.8）mm，较影像学测量值均小。

浸润前及微浸润病变组（AAH/AIS/MIA）和浸润病变组（IAC）之间的三维最大径、平均CT值、中位CT值、10%位数CT值、90%位数CT值的差异均有统计学意义（P<0.05）（表3）。对三维最大径和平均CT值进行ROC曲线分析结果显示（图3A），曲线下面积分别为0.917（95%CI: 0.809-1.000）（P=0.002）和0.885（95%CI: 0.764-1.000）（P=0.004），界值分别为14.4 mm和-495.7 HU，灵敏度均为100.0%，特异度均为73.1%。

**表3:** 浸润前及微浸润病变组与浸润病变组的单因素分析

Feature	AAH/AIS/MIA	IAC	t	P
2D diameter (mm)	10.0±3.4	17.7±8.8	2.097	0.086
3D diameter (mm)	11.7±3.9	22.1±8.0	3.087	0.024
Mean CT value (HU)	-562.3±130.8	-350.8±115.7	3.636	0.001
Median CT value (HU)	-585.5±149.2	-369.0±135.1	3.253	0.003
10% percentile CT value (HU)	-779.6±67.7	-690.3±79.7	2.778	0.010
90% percentile CT value (HU)	-320.6±201.6	-24.7±167.7	3.328	0.002

IAC: invasive adenocarcinoma; MIA: minimally invasive adenocarcinoma; AIS: adenocarcinoma in situ; AAH: atypical adenomatous hyperplasia.

**图3:**
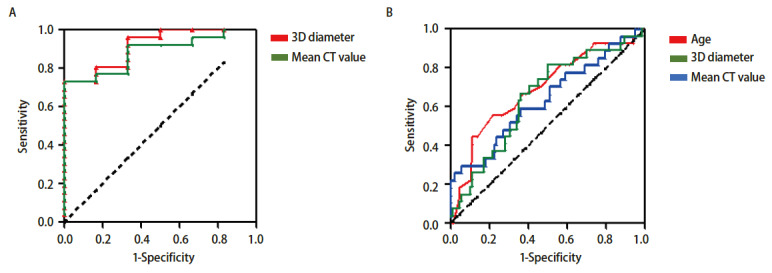
ROC曲线。 A：切除病变的ROC曲线；B：随访GGNs的ROC曲线。

### 2.4 影响GGNs增长的危险因素

单因素分析结果显示（表1，表2）增长组与稳定组的形态规则与否、边缘是否光整，有无分叶征、毛刺征、空泡征、年龄、基线直径和CT值的差异均具有统计学意义。多因素Logistic回归分析结果表明年龄（OR=1.075; 95%CI: 1.015-1.138; P=0.013）和平均CT值（OR=1.009; 95%CI: 1.001-1.016; P=0.021）是促进结节增长的危险因素。

ROC曲线分析结果（图3B）提示年龄≥63岁（曲线下面积为0.691，95%CI: 0.572-0.809，P=0.003，灵敏度为55.6%，特异度为78.4%），基线三维最大径≥9.2 mm（曲线下面积为0.645，95%CI：0.527-0.762，P=0.023，灵敏度为81.5%，特异度为50.0%），平均CT值≥-507.8 HU（曲线下面积为0.637，95%CI：0.510-0.763，P=0.032，灵敏度均为29.6%，特异度均为94.3%）的GGNs出现增长的可能性更大。

## 3 讨论

目前，肺癌仍是全球发病率和死亡率最高的恶性肿瘤^[18]^，一直以来东西方都致力于肺癌筛查来实现早诊断早治疗^[19,20]^。自低剂量CT平扫在肺癌筛查中普遍开展以来，肺癌相关死亡率明显降低，同时无症状GGNs的检出率也日益增高。CT影像表现为短暂出现的GGNs多为炎性病变，表现为持续存在的GGNs具有鲜明的惰性生物学行为，进展缓慢，尤其是pGGNs，甚至几年都不会发生变化。pGGNs者常提示病理类型为AAH或AIS，具有良好预后，但其仍具有恶变潜能；PSNs较pGGNs生长要快且恶性程度更高，当结节增长速度变快时，高度提示恶性可能^[21,22]^。从病理学角度出发GGNs的生长遵循自AAH向AIS、MIA直至IAC发展的规律。同时已有研究^[23]^证实浸润前病变和贴壁为主的浸润性腺癌（lepidic predominant adenocarcinoma, LPA）的5年无复发生存率均为100%，因而对GGNs进行科学合理管理可为降低肺癌发生风险做出重大贡献，同时为减轻患者的精神压力和经济负担及避免诊疗不足或过度这一目的，掌握GGNs的生长发展规律就显得尤为重要。

本研究随访过程中27例（23.5%）出现增长，单因素分析结果显示增长组与稳定组的形态边缘特征、分叶征、空泡征、毛刺征、空气支气管征、基线直径大小均有统计学意义，多因素Logistic回归分析结果表明高龄与高CT值为促进结节增长的危险因素。本研究利用了3D Slicer软件对结节进行了三维立体测量，发现增长组与稳定组的基线表面积、体积、质量的差异并不明显，这可能与我们的研究主要采用更具有临床实践意义的直径变化作为结节增长的判定标准有关，但测量值对比可发现随访终末时中位值（表面积169.3 mm^2^，体积178.9 mm^3^，质量59.8 mg）较基线时（表面积117.3 mm^2^，体积97.6 mm^3^，质量32.3 mg）均明显增大。

Kakinuma等^[7]^于2009年开展的首项多中心前瞻性随访研究结果表明1,229例GGNs中仅1%病理证实为浸润性腺癌且均为混合GGNs，结节初始最大径是预测结节增长的主要因素，而肺恶性肿瘤史不作为其风险因素，并指出从避免过度治疗的角度出发，实性成分的出现可能不是手术干预的合适指征。以往有研究^[24]^表明，恶性肿瘤家族史可增加患者结节的恶性风险以及结节增长风险。Kim等^[25]^对肺切除术后患者GGNs的随访分析显示仅实性成分出现是其增长的预测因素，且呈惰性生长过程，恶性肿瘤家族史相关性差异无统计学意义。此类患者结节仍可采用术前结节相同管理模式，避免早期干预而降低患者肺功能，降低生活质量。Lee等^[26]^则认为空泡征、恶性肿瘤史而非肺恶性肿瘤史以及新增实性成分是影响GGNs增长的重要危险因素。

目前有指南^[27]^推荐不超过5 mm的pGGNs无需随访，Lee等^[26]^在对稳定存在5年以上的GGNs的随访研究中发现27例（13%）出现了增长，其中约95%基线直径<6 mm，随访8.5年后增长3.2 mm。因此更推荐对直径较小的pGGNs能进行年度低剂量CT随访。本研究对基线三维最大径进行ROC分析，得出三维最大径曲线下面积为0.645，界值9.19 mm，灵敏度为81.5%，特异度为50.0%。Cho等^[28]^研究发现3D最大径>8 mm的pGGNs在随访期间更易出现增长。Shi等^[29]^对101例pGGNs的回顾性研究结果显示三维最大径的截断值为10.2 mm。目前大量对二维直径的研究^[29,30]^结果显示基线直径≥10 mm的pGGNs出现增长的可能性较大。

本研究ROC分析结果显示，平均CT值曲线下面积为0.637，界值为-507.8 HU，灵敏度为29.6%，特异度为94.3%。与以往报道文献结果存在差异，Eguchi等^[31]^报道平均CT值界值-670 HU，Tamura等^[32]^研究CT值预测结节进展的界值为-677 HU。这可能与CT值测量易受测量方法和患者扫描时呼吸状态影响有关。

对32例切除GGNs的研究结果显示区别浸润病变与浸润前病变的三维最大径界值为14.4 mm，平均CT值为-495.7 HU，与既往文献^[33,34]^报道结果相类似。本研究对切除结节二维直径和三维直径的结果比较显示差异具有统计学意义（P<0.001）。切除结节中有5例因二维直径出现增长而进行干预，术后病理结果均为MIA，但其三维直径未显示明显增长。因此，认为采用三维测量值判断增长更为准确。

本研究利用3D Slicer软件对结节表面积和体积进行测量，由于GGNs大多不是规则的球体，采用长径和垂直径的方法计算结节体积容易引起较大误差，人工智能辅助结节分割的方式测量结节体积，能减少测量误差，结果准确性更高。计算结节质量^[35]^结果表明增长组和稳定组表面积、体积和质量的差异均无统计学意义（P>0.05），而浸润前病变组与浸润病变组表面积（P=0.001）、体积（P=0.002）、质量（P=0.001）差异均有统计学意义。中位体积倍增时间为822 d，中位质量倍增时间为1,007 d，本研究结果显示质量倍增之间值较体积倍增时间更长，可能与纳入结节密度变化较慢相关。

在我国，发现混杂成分增大或新的实性成分出现往往不会待其增长至14 mm，而是及早进行干预。目前手术方式主要以楔形切除及肺段切除为主。中央带和外周带所选择的手术方式不同，中央带其多采用肺叶切除，手术创伤相较于肺段或楔形切除要大，因此中央带更建议在安全期内延长随访时间。倍增时间较长的GGNs频繁随访会给患者带来不必要的辐射伤害和焦虑情绪，规范随访，不会误诊漏诊，避免手术创伤提前，提高患者生活质量。

本研究存在一定的局限性：首先，数据来源于单一中心，存在选择偏倚，样本量也较小，纳入研究对象的男女比例失衡也可能与肺腺癌在不吸烟女性中发生概率更高有关；其次，结节进展可能相关恶性肿瘤病史、家族史、职业及心理情绪等因素未纳入分析；再者，GGNs多数形状不规则，边界不清晰，轮廓勾画存在误差；最后，未将GGNs的体积或质量变化纳入评价标准。

综上所述，GGNs呈惰性生长过程，三维立体测量可更准确地判断结节增长，在随访过程中的应用价值更大。年龄≥63岁、基线三维最大径≥9.2 mm、平均CT值≥-507.8 HU的GGNs出现增长的可能性更大，但多数结节出现进展后仍预后良好，因此适当延长随访时间，避免过早手术干预是安全的。
